# Anticipation of cognitive conflict is reflected in microsaccades: Evidence from a cued-flanker task

**DOI:** 10.16910/jemr.12.6.3

**Published:** 2019-01-24

**Authors:** Mario Dalmaso, Luigi Castelli, Giovanni Galfano

**Affiliations:** University of Padova, Italy

**Keywords:** microsaccades, eye movements, eye tracking, attention, cognitive conflict, cued-flanker task

## Abstract

Microsaccade frequency has recently been shown to be sensitive to high-level cognitive processes such as attention and memory. In the present study we explored the effects of anticipated cognitive conflict. Participants were administered a variant of the flanker task, which is known to elicit cognitive interference. At the beginning of each trial, participants received a colour cue providing information about the upcoming target frame. In two thirds of the trials, the cue reliably informed the participants that in the upcoming trial the flankers either matched the central target letter or not. Hence, participants could accurately anticipate whether cognitive conflict would arise or not. On neutral trials, the cue provided no useful information. The results showed that microsaccadic rate time-locked to cue onset was reduced on trials in which an upcoming cognitive conflict was expected. These findings provide new insights about top-down modulations of microsaccade dynamics.

## Introduction

Prolonged fixation is usually associated with the unaware production of rapid and tiny eye movements called microsaccades, which are involved in perceptual processing (e.g., [1, 2, 3, 4]). Typically, microsaccadic rate baseline is around 1-2 Hz but, after a perceptual transient, this rate production is characterized by 1) an inhibition phase, followed by a 2) rebound phase and a 3) return to the baseline (e.g., [5]).

Interestingly, evidence is quickly accumulating showing that microsaccadic dynamics can be shaped even by higher order mechanisms, such as orienting of attention (e.g., [5, 6, 7]) or conscious perception [8]. Working memory load and mental counting are also known to impact on microsaccade generation: In this context, more demanding processing conditions (i.e., higher load) have been associated with a decrement in microsaccadic rate as compared to less demanding processing conditions (i.e., lower load; e.g., [9, 10, 11, 12]).

A greater decrement in microsaccadic rate has been reported even when participants were asked to prepare for a manual response to an upcoming target as compared to a condition in which they were asked to look at the target passively [13]. In a similar vein, less microsaccades have also been observed when participants were asked to prepare for an anti-saccade (i.e., an eye movement performed towards the location opposite to the target) as compared to a pro-saccade (i.e., an eye movement performed towards a target [14, 15, 16]). Since anti-saccades require to inhibit the prepotent tendency to look at the target while pro-saccades rely on more automatic processes (e.g., [17]), the mechanisms implied in pro-/anti-saccade preparation might be interpreted as reflecting cognitive control (see also [18]). Taken together, these studies lead to two main conclusions. First, they invite to consider microsaccades as a direct oculomotor index to track preparatory mechanisms for upcoming events. Second, they suggest that microsaccades could even reflect the degree of cognitive control implicated to properly deal with an upcoming event.

The aim of the present study was to directly explore the potential impact of expected conflict on microsaccadic dynamics by employing a version of the flanker task, a widely-employed paradigm for the study of cognitive control mechanisms (e.g., [19]; see also [20]). In a typical flanker task, participants are asked to discriminate a central target letter (e.g., “H”) flanked by non-target letters that can be either identical (i.e., “HHH”; congruent condition) or different (e.g., “SHS”; incongruent condition) with respect to the target letter. Smaller latencies and greater accuracies are generally reported in the congruent than in the incongruent condition, indicating that the two conditions differ in terms of difficulty (i.e., the congruent condition is easier than the incongruent condition). Here, expected conflict was manipulated by employing a cued version of the flanker task (see [21], Experiment 3) in which three different visual cues – provided at the beginning of the trial – informed participants regarding the nature of the upcoming flanker stimulus. Two cues were 100% informative, namely one cue always predicted a congruent flanker stimulus while another cue always predicted an incongruent flanker stimulus. The third cue was uninformative (i.e., neutral), since congruent and incongruent stimuli might appear with the same probability (i.e., 50%).

In line with previous studies addressing different issues related to cognitive control (e.g., [15, 16]), we hypothesized that the anticipation of cognitive conflict should be reflected in microsaccadic rate. In particular, we expected that the informative cue predicting an incongruent flanker stimulus would be associated with a reduction in microsaccadic rate as compared to the informative cue predicting a congruent flanker stimulus. This would be consistent with the idea that anticipation of a higher cognitive effort is associated with a decrease in microsaccadic rate.

## Methods

### Participants

Thirty naïve students (*Mean age* = 23.6 years, *SD* = 2.06, 4 males, 3 left-handed) took part on a voluntary basis. Their vision was normal or corrected to normal with lenses. The Ethics Committee for Psychological Research at the University of Padova approved the study, that was conducted in accordance with the Declaration of Helsinki. A written informed consent was obtained from all participants.

### Apparatus

An EyeLink 1000 Plus (SR Research Ltd, Ottawa, Canada) recorded binocular eye movements (see [22]) at 500 Hz. Participants sat 65 cm away from a 24-inch monitor (1280 × 1024 pixels, 120 Hz) and a chinrest was used to prevent head movements. Timing and stimuli presentation were handled with Experiment Builder (SR Research Ltd, Ottawa, Canada). Room luminance and screen background (grey coloured; R = 180, G = 180, B = 180) were kept constant throughout the experiment and they were identical for all participants.

### Stimuli and procedure

**Figure 1.**
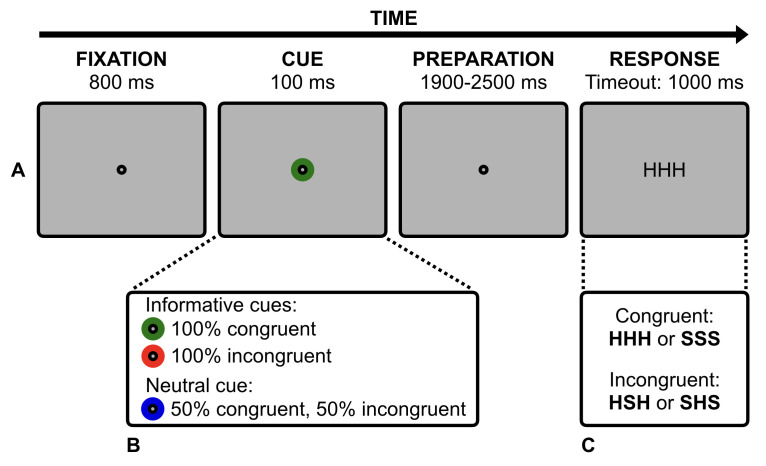
Illustration of the cued-flanker task. Participants maintained fixation on the central spot for the whole duration of the trial. The central cue (i.e., the coloured ring), provided information concerning the upcoming flanker stimulus, and a speeded manual response was required to decide whether the central target letter was either “H” or “S”.

A nine-point calibration was followed by a validation procedure. Before each trial, a drift checking was performed, in which participants looked at a central black dot (diameter: 0.4°) and then the experimenter initiated the trial through the host PC. This allowed us to ensure that participants were looking at the centre of the screen. A positive drift checking was associated with a tone, that informed participants of the forthcoming trial start. Each trial started with a central black spot (diameter: 0.4°; see Figure 1, Panel A, fixation frame) for 800 ms. Then, the fixation spot was surrounded by a coloured ring (diameter: 1.2°) that acted as a cue (Figure 1, Panel A, cue frame). The ring could be coloured in green, blue or red. The three colours were matched for luminance (60 cd/m^2^; OptiCAL luminance metre device, Cambridge Research Systems). Participants were informed that green and red rings were 100% predictive (i.e., the cue was informative) of a congruent (i.e., HHH or SSS) and an incongruent (i.e., HSH or SHS) flanker stimulus, respectively. The blue ring, instead, was only 50% predictive (i.e., the cue was neutral) of either a congruent or an incongruent flanker stimulus (i.e., HHH, SSS, HSH or SHS; Figure 1, Panel B). After 100 ms, the coloured ring disappeared, and the fixation spot remained on the screen for a variable temporal interval of 1900-2500 ms (100-ms steps; Figure 1, Panel A, preparation frame). Then, the fixation spot disappeared and the flanker stimulus (.98° width × .37° height; 16-point Arial) appeared in black at the centre of the screen (Figure 1, Panel A, response frame). Flanker stimuli were composed of H and S letters (i.e., HHH and SSS as for the congruent stimuli, HSH and SHS as for the incongruent stimuli; Figure 1, Panel C). The flanker stimulus remained visible until a response was made or after a 1000-ms timeout, whichever came first. Participants were instructed to press one of two buttons to classify – as fast and accurate as possible – the central letter as an H or an S. The association between response buttons and target letters was counterbalanced across participants. Finally, a first visual feedback (800 ms) informed the participants about their performance (i.e., “ok” for correct responses, “no” for wrong responses, “faster” for no responses) and then a second visual feedback (800 ms) invited the participants to blink, if needed. Participants were also instructed to maintain their eyes at the centre of screen and to avoid blinks for the whole duration of the trial, otherwise a 800-ms central error feedback appeared and the trial was aborted and appended at the end of the experimental session. This allowed us to collect a reasonable number of blink-free epochs while avoiding an excessive duration of the experiment. A practice block composed of 12 randomly-presented trials was followed by 300 randomly-presented experimental trials. The three cues were presented for an equal number of trials. A short break was allowed every 50 trials. The whole experiment lasted about 1 hour.

## Results

### Manual responses

Trials in which participants committed an error (3.72% of trials) or did not provide a response (1.87% of trials) were removed and analysed separately.

Three repeated-measures ANOVAs with Cue (2: informative vs. neutral) and Congruency (2: congruent vs. incongruent) as within-participants factors were employed to analyse the percentage of errors, the percentage of missed responses and median reaction times (RTs) of correct trials.

As for errors, the main effect of Congruency was significant, *F*(1, 29) = 11.193, *p* = .002, *η* ^*2*^_*p *_ = .278, due to fewer errors on congruent trials (*M* = 2.65%, *SE* = .503) than on incongruent trials (*M* = 4.75%, *SE* = .801). No other significant results emerged (*p*s > .058).

As for missed responses, the main effect of Congruency was significant, *F*(1, 29) = 6.696, *p* = .014, *η* ^*2*^_*p *_ = .188, due to fewer missed responses on congruent trials (*M* = 1.59%, *SE* = .548) than on incongruent trials (*M* = 2.32%, *SE* = .623). No other significant results emerged (*p*s > .433).

**Figure 2.**
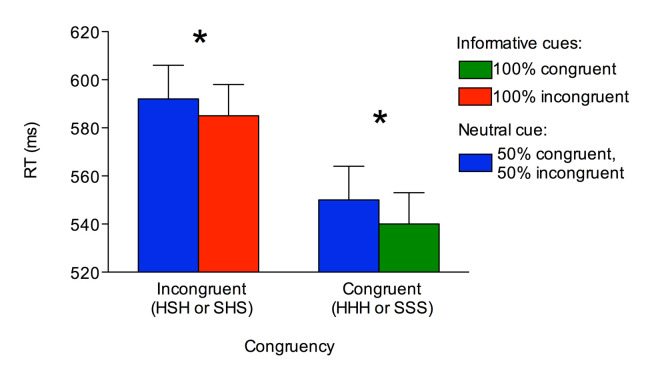
Manual reaction times reported for each experimental condition. Asterisks denote *p* < .05.

As for RTs, the main effect of Cue was significant, *F*(1, 29) = 8.740, *p* = .006, *η* ^*2*^_*p *_ = .232, due to lower RTs for informative cues (*M* = 562 ms, *SE* = 12.96) than neutral cues (*M* = 571 ms, *SE* = 14), as well as the main effect of Congruency, *F*(1, 29) = 81.588, *p* < .001, *η* ^*2*^_*p *_ = .738, due to lower RTs on congruent trials ( *M* = 545 ms, *SE* = 13.67) than on incongruent trials ( *M* = 589 ms, *SE* = 13.59). The interaction was non-significant (*p* = .443). Nevertheless, for completeness, one-tailed comparisons (see also [21]) between congruent and incongruent trials were performed separately for each cue. These confirmed that, regardless of congruency, RTs were lower for informative than neutral cues (*p*s < .038; see Figure 2)

Overall, these results indicated that participants adapted their behaviour in accordance with the cue, in line with previous studies that employed a cued-flanker task (e.g., [21, 23]).

### Microsaccades

Binocular microsaccades were extracted by employing the algorithm proposed by Engbert and Kliegl [5] adapted for a 500 Hz sampling rate. The velocity threshold was set to λ = 6 and the minimum duration threshold was set to 3 samples. Only microsaccades with a maximum amplitude of 1° were considered (see [3]). Only trials in which manual response was correct were analysed.

**Figure 3.**
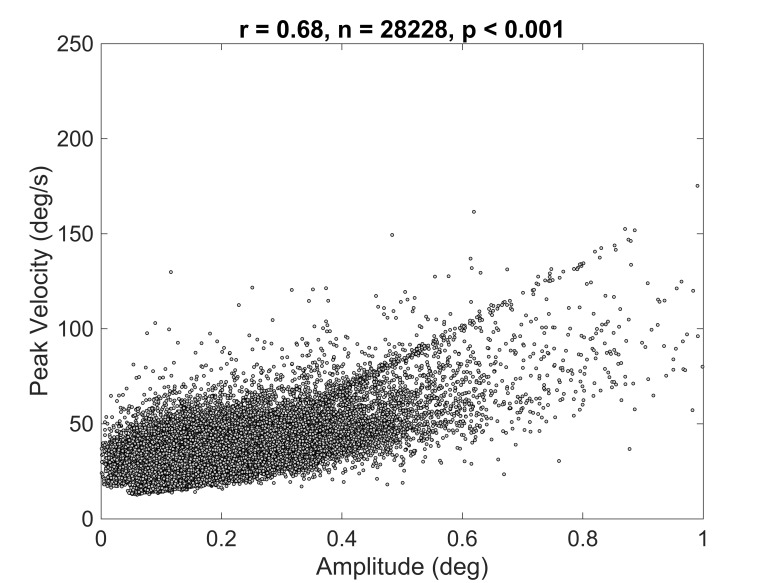
Correlation between microsaccadic amplitude and peak velocity.

First, we verified that presence of the so-called main sequence (see [24]), namely a positive relationship between microsaccadic amplitude and peak velocity. This was confirmed by a correlational analysis, *r* = .68, *p* < .001, suggesting that microsaccades were identified correctly (Figure 3).

**Figure 4.**
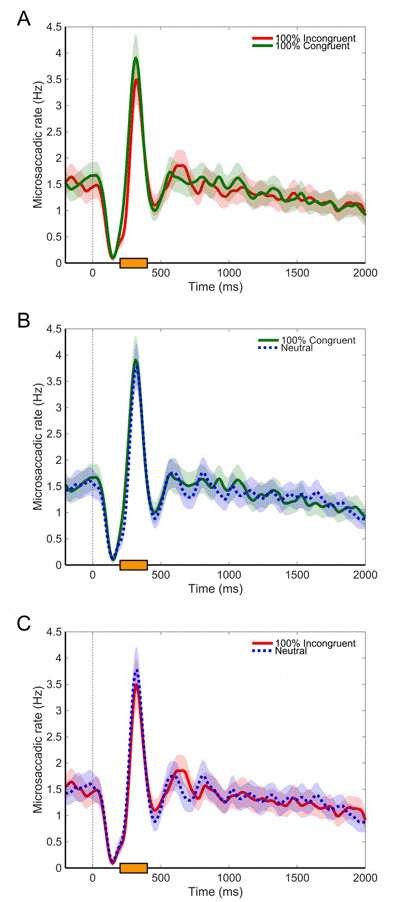
Mean microsaccadic rate, computed within a time window starting from cue onset (i.e., t = 0) and ending at the flanker stimulus onset (i.e., t = 2000). Shaded areas are SEM. Panel A depicts the 100% congruent vs. 100% incongruent comparison; Panel B depicts the 100% congruent vs. neutral comparison; Panel C depicts the 100% incongruent vs. neutral comparison. The orange rectangle indicates the 200-400 ms temporal window in which a difference between conditions emerged.

After that, microsaccadic rate was computed within a 2000-ms temporal epoch time-locked to cue onset, separately for each participant and Cue (100% congruent, 100% incongruent, neutral), and then averaging the data across participants. As shown in all panels of Figure 4, cue onset (i.e., t = 0) led to a microsaccadic inhibition phase that was followed by a rebound phase and a return to the baseline, in line with previous evidence (e.g., [5, 25]). Then, ten fdr-corrected comparisons between mean microsaccadic rate for each combination resulting from Cue factor (i.e., 100% congruent vs. 100% incongruent, see Panel A; 100% congruent vs. neutral, see Panel B; 100% incongruent vs. neutral, see Panel C) were performed through a 200-ms moving window starting at cue onset (for a similar approach see also [9, 12, 15]). As for the 100% congruent vs. 100% incongruent comparison, the only significant difference emerged within the 200-400 ms time window, *t*(29) = 3.718, *p* < .001, which seems to correspond to the rebound phase, based on visual inspection of the data. No other significant results emerged (*p*s > .218). As for the 100% congruent vs. neutral comparison, no significant differences emerged (*p*s > .491), as well as for the 100% incongruent vs. neutral comparison (*p*s > .241). Microsaccadic rate within the 200-400 ms time window was further analysed through an ANOVA analysis with Cue (100% congruent, 100% incongruent, neutral) as within-participant factor. The main effect was significant, *F*(2, 58) = 7.548, *p* = .001, *η* ^*2*^_*p *_ = .207. Two-tailed fdr-corrected comparisons indicated that the 100% incongruent cue led to a greater decrement in microsaccadic rate as compared to both the 100% congruent cue, *t*(29) = 3.718, *p* = .0027, and the neutral cue, *t*(29) = 2.375, *p* = .036, while the comparison between the 100% congruent and the neutral cue was non-significant, *t*(29) = 1.423, *p* = .165. These comparisons were further analysed by computing Bayes Factors (BF_10_), to assess whether the current data provided more support for the alternative hypothesis (i.e., a difference between two conditions) or the null hypothesis (i.e., no difference between two conditions). According to Jeffreys [26], a very strong supporting evidence for the alternative hypothesis emerged from the comparison between the 100% incongruent cue and the 100% congruent cue, *BF*_*10*_ = 37.999, whereas this evidence was weaker for the comparison between the 100% incongruent cue and the neutral cue, *BF*_*10*_ = 2.139. Finally, evidence supporting the null hypothesis emerged from the comparison between the 100% congruent cue and the neutral cue, *BF*_*10*_ = .483.

## Discussion

In this study we investigated whether anticipation of cognitive conflict is reflected in microsaccadic rate. To this aim, we employed a cued-flanker task in which a cue, provided at the beginning of each trial, informed participants regarding the nature of the upcoming flanker stimulus. On two thirds of the trials, the cue was 100% predictive of either a congruent or an incongruent flanker stimulus (i.e., informative cue). On the remaining trials, the cue did not predict the nature of the upcoming flanker stimulus (i.e., neutral cue). Manual response analyses confirmed that participants were able to adjust their behaviour in accordance with the cue, since RTs were overall smaller for predictive than neutral cues, a result in line with previous studies (e.g., [21, 23]). More interestingly, in line with our hypotheses, microsaccadic rate was lower when participants were expecting a cognitive conflict (i.e., the cue was predictive of a 100% incongruent trial), and this was particularly evident in the comparison with the condition in which participants were expecting no conflict (i.e., the cue was predictive of a 100% congruent trial). Interestingly, this difference in microsaccadic rate emerged during the rebound phase, a pattern that aligns with previous studies in which cognitive load was manipulated (e.g., [9, 10, 12]) and – more in general – with the idea that the rebound phase might be more revealing of an impact of higher-order mechanisms as compared to the inhibition phase (see also [27]). This might occur because, while the inhibition phase occurs early in time – likely reflecting a physiological response to a visual input – the rebound phase is a later component and therefore more permeable to different cognitive factors. However, the actual mechanisms underlying this biphasic pattern is still debated (e.g., [28]).

In recent years, an increasing number of studies has reported that more demanding cognitive tasks are often associated with a decrement in microsaccadic rate (e.g., [9, 11, 15, 16, 29, 30, 31]; but see also [32, 33, 34]). In accordance with Siegenthaler [11], this decrement would be caused by a poorer fixational activity due to working memory load. The same rationale could be also applied in the present context, since it is known that working memory load can shape performance during a flanker task (e.g., [35]).

Tentatively, a further evidence for a potential link between cognitive control and microsaccades might be found even under a neuroanatomical perspective. Indeed – on the one hand – both psychophysical (see [27]) and neurophysiological (see [36]) studies provided converging evidence indicating that microsaccades would be generated in the superior colliculus (SC), and a recent study [37] showed that also the frontal eye fields (FEF) seem to play a role in microsaccadic generation. On the other hand, the main brain region recruited in a flanker task would be the anterior cingulate, but prefrontal areas placed in proximity of the FEF would be also involved (see [38]), thus suggesting a potential overlap with the neural substrates of microsaccades.

Taken together, the results of this study suggest that anticipating cognitive conflict can shape microsaccadic rate. A similar modulation has previously been reported in studies in which participants were asked to prepare for a pro-saccade as compared to an anti-saccade [14, 15, 16]. Even if both the pro-/anti-saccade task and the flanker task can be employed to investigate cognitive control (e.g., [18, 39]), these two paradigms call into play different cognitive mechanisms. Indeed, while anti-saccades require the control of attention away from the target to inhibit a prepotent automatic response (see [17]), the flanker task requires to allocate attentional resources on the target to discard the interfering information provided by distracting stimuli (see [19]). In addition, the time window of interest in the present study was that in between cue onset and flanker stimulus onset (i.e., we were interested in addressing whether microsaccades can reflect anticipation of conflict rather than shielding against interference). Moreover, while the pro-/anti-saccade task employs eye movements, the flanker task is typically based on manual responses. Hence, these two tasks can provide complementary but distinct information concerning the potential relationship between cognitive control and microsaccades. The dissimilar nature of the two tasks might also explain why the previous studies on cognitive control reported differences in microsaccades that were apparently present throughout the preparatory period [14, 15, 16], while here a difference emerged only around the rebound phase. Nevertheless, since the potential impact of cognitive control on microsaccade generation remains widely unexplored, future work is necessary to shed light on this kind of top-down modulations. For instance, different cognitive control tasks could be employed to generate microsaccadic results. These, in turn, could be used to develop computational models, which are particularly suitable to test novel hypotheses (e.g., [40]).

The ability to properly plan for – and then execute – a certain behavior is an essential ability to successfully navigate within complex environments. In this regard, microsaccades could be considered as a direct, non-invasive tool to track ongoing preparatory mechanisms that might be employed in different contexts, such as everyday activities (e.g., driving [32, 41]), human-computer interactions (e.g., air traffic control [34]) or even clinical assessment (e.g., ADHD [42]).

## Ethics and Conflict of Interest

The authors declare that the contents of the article are in agreement with the ethics described in http://biblio.unibe.ch/portale/elibrary/BOP/jemr/ethics.html and that there is no conflict of interest regarding the publication of this paper.

## Acknowledgements

We are grateful to Professor Reinhold Kliegl and an anonymous reviewer for helpful comments on a previous version of this work. We also wish to thank Ilaria Schettino for her assistance in data collection.

## References

[b32] Benedetto, S. , Pedrotti, M. , & Bridgeman, B. ( 2006). Microsaccades and exploratory saccades in a naturalistic environment. Journal of Eye Movement Research, 4, 1–10. 1995-8692

[b13] Betta, E. , & Turatto, M. ( 2006). Are you ready? I can tell by looking at your microsaccades. Neuroreport, 17, 1001–1004. 10.1097/01.wnr.0000223392.82198.6d 0959-4965 16791092

[b6] Betta, E. , Galfano, G. , & Turatto, M. ( 2007). Microsaccadic response during inhibition of return in a target-target paradigm. Vision Research, 47, 428–436. 10.1016/j.visres.2006.09.010 0042-6989 17087989

[b39] Botvinick, M. M. , Braver, T. S. , Barch, D. M. , Carter, C. S. , & Cohen, J. D. ( 2001). Conflict monitoring and cognitive control. Psychological Review, 108, 624–652. 10.1037/0033-295X.108.3.624 0033-295X 11488380

[b33] Chen, Y. , Martinez-Conde, S. , Macknik, S. L. , Bereshpolova, Y. , Swadlow, H. A. , & Alonso, J.-M. ( 2008). Task difficulty modulates the activity of specific neuronal populations in primary visual cortex. Nature Neuroscience, 11, 974–982. 10.1038/nn.2147 1097-6256 18604204 PMC2553692

[b1] Collewijn, H. , & Kowler, E. (2008).The significance of microsaccades for vision and oculomotor control. Journal of Vision (Charlottesville, Va.),8,20.1– 21. 10.1167/8.14.20 1534-7362 19146321 PMC3522523

[b23] Correa, A. , Rao, A. , & Nobre, A. C. (2009).Anticipating conflict facilitates controlled stimulus-response selection. Journal of Cognitive Neuroscience,21,1461–1472. 10.1162/jocn.2009.21136 0898-929X 18823248 PMC4152723

[b9] Dalmaso, M. , Castelli, L. , Scatturin, P. , & Galfano,G. (2017).Working memory load modulates microsaccadic rate. Journal of Vision (Charlottesville, Va.),17,6. 10.1167/17.3.6 1534-7362 28278311

[b14] Dalmaso,M. , Castelli,L. , & Galfano,G. (2019). (in press). Microsaccadic rate and pupil size dynamics in pro-/anti-saccade preparation: The impact of intermixed vs. blocked trial administration. Psychological Research.Advance online publication. 10.1007/s00426-018-01141-7 0340-0727 30603866

[b34] Di Stasi,L. L. , McCamy,M. B. , Catena,A. , Macknik,S. L. , Cañas,J. J. , & Martinez-Conde,S. (2013).Microsaccade and drift dynamics reflect mental fatigue. The European Journal of Neuroscience,38,2389–2398. 10.1111/ejn.12248 0953-816X 23675850

[b41] Di Stasi,L. L. , McCamy,M. B. , Pannasch,S. , Renner,R. , Catena,A. , Cañas,J. J. , Velichkovsky,B. M. , & Martinez-Conde,S. (2015).Effects of driving time on microsaccadic dynamics. Experimental Brain Research,233,599–605. 10.1007/s00221-014-4139-y 0014-4819 25417191

[b5] Engbert,R. , & Kliegl,R. (2003).Microsaccades uncover the orientation of covert attention. Vision Research,43,1035–1045. 10.1016/S0042-6989(03)00084-1 0042-6989 12676246

[b2] Engbert,R. (2006).Microsaccades: A microcosm for research on oculomotor control, attention, and visual perception. Progress in Brain Research,154,177– 192. 10.1016/S0079-6123(06)54009-9 0079-6123 17010710

[b40] Engbert,R. (2012).Computational modeling of collicular integration of perceptual responses and attention in microsaccades. The Journal of Neuroscience : The Official Journal of the Society for Neuroscience,32,8035–8039. 10.1523/JNEUROSCI.0808-12.2012 0270-6474 22674278 PMC6620943

[b19] Eriksen,B. A. , & Eriksen,C. W. (1974).Effects of noise letterls upon the identification of a target letter in a nonsearch task. Perception & Psychophysics,16,143–149. 10.3758/BF03203267 0031-5117

[b38] Fan,J. , McCandliss,B. D. , Fossella,J. , Flombaum,J. I. , & Posner,M. I. (2005).The activation of attentional networks. NeuroImage,26,471–479. 10.1016/j.neuroimage.2005.02.004 1053-8119 15907304

[b42] Fried,M. , Tsitsiashvili,E. , Bonneh,Y. S. , Sterkin,A. , Wygnanski-Jaffe,T. , Epstein,T. , & Polat,U. (2014).ADHD subjects fail to suppress eye blinks and microsaccades while anticipating visual stimuli but recover with medication. Vision Research,101,62–72. 10.1016/j.visres.2014.05.004 0042-6989 24863585

[b10] Gao,X. , Yan,H. , & Sun,H.-J. (2015).Modulation of microsaccade rate by task difficulty revealed through between- and within-trial comparisons. Journal of Vision (Charlottesville, Va.),15,1–15. 10.1167/15.3.3 1534-7362 25740876

[b21] Gratton,G. , Coles,M. G. , & Donchin,E. (1992).Optimizing the use of information: Strategic control of activation of responses. Journal of Experimental Psychology. General,121,480–506. 10.1037/0096-3445.121.4.480 0096-3445 1431740

[b7] Hafed,Z. M. , & Clark,J. J. (2002).Microsaccades as an overt measure of covert attention shifts. Vision Research,42,2533–2545. 10.1016/S0042-6989(02)00263-8 0042-6989 12445847

[b36] Hafed,Z. M. , Goffart,L. , & Krauzlis,R. J. (2009).A neural mechanism for microsaccade generation in the primate superior colliculus. Science,323,940–943. 10.1126/science.1166112 0036-8075 19213919 PMC2655118

[b28] Hafed,Z. M. , & Ignashchenkova,A. (2013).On the dissociation between microsaccade rate and direction after peripheral cues: Microsaccadic inhibition revisited. The Journal of Neuroscience : The Official Journal of the Society for Neuroscience,33,16220–16235. 10.1523/JNEUROSCI.2240-13.2013 0270-6474 24107954 PMC6618351

[b15] Hermens,F. , Zanker,J. M. , & Walker,R. (2010).Microsaccades and preparatory set: A comparison between delayed and immediate, exogenous and endogenous pro- and anti-saccades. Experimental Brain Research,201,489–498. 10.1007/s00221-009-2061-5 0014-4819 19946771

[b22] Hermens,F. (2015).Dummy eye measurements of microsaccades: Testing the influence of system noise and head movements on microsaccade detection in a popular video-based eye tracker. Journal of Eye Movement Research,8,1–17.1995-8692

[b18] Hutton,S. B. (2008).Cognitive control of saccadic eye movements. Brain and Cognition,68,327–340. 10.1016/j.bandc.2008.08.021 0278-2626 19028265

[b26] Jeffreys,H. (1961).Theory of probability (3rd ed.).Oxford University Press.

[b29] Krejtz,K. , Duchowski,A. T. , Niedzielska,A. , Biele,C. , & Krejtz,I. (2018).Eye tracking cognitive load using pupil diameter and microsaccades with fixed gaze. PLoS One,13,e0203629. 10.1371/journal.pone.0203629 1932-6203 30216385 PMC6138399

[b30] Lange,E. B. , Zweck,F. , & Sinn,P. (2017).Microsaccade-rate indicates absorption by music listening. Consciousness and Cognition,55,59–78. 10.1016/j.concog.2017.07.009 1053-8100 28787663

[b35] Lavie,N. , Hirst,A. , de Fockert,J. W. , & Viding,E. (2004).Load theory of selective attention and cognitive control. Journal of Experimental Psychology. General,133,339–354. 10.1037/0096-3445.133.3.339 0096-3445 15355143

[b3] Martinez-Conde,S. , Otero-Millan,J. , & Macknik,S. L. (2013).The impact of microsaccades on vision: Towards a unified theory of saccadic function. Nature Reviews. Neuroscience,14,83– 96. 10.1038/nrn3405 1471-003X 23329159

[b20] Miller,J. (1991).The flanker compatibility effect as a function of visual angle, attentional focus, visual transients, and perceptual load: A search for boundary conditions. Perception & Psychophysics,49,270–288. 10.3758/BF03214311 0031-5117 2011464

[b17] Munoz,D. P. , & Everling,S. (2004).Look away: The anti-saccade task and the voluntary control of eye movement. Nature Reviews. Neuroscience,5,218–228. 10.1038/nrn1345 1471-003X 14976521

[b31] Pastukhov,A. , & Braun,J. (2010).Rare but precious: Microsaccades are highly informative about attentional allocation. Vision Research,50,1173–1184. 10.1016/j.visres.2010.04.007 0042-6989 20382176

[b37] Peel,T. R. , Hafed,Z. M. , Dash,S. , Lomber,S. G. , & Corneil,B. D. (2016).A Causal role for the cortical frontal eye fields in microsaccade deployment. PLoS Biology,14,e1002531. 10.1371/journal.pbio.1002531 1544-9173 27509130 PMC4980061

[b4] Poletti,M. , & Rucci,M. (2016).A compact field guide to the study of microsaccades: Challenges and functions. Vision Research,118,83– 97. 10.1016/j.visres.2015.01.018 0042-6989 25689315 PMC4537412

[b27] Rolfs,M. , Kliegl,R. , & Engbert,R. (2008).Toward a model of microsaccade generation: The case of microsaccadic inhibition. Journal of Vision (Charlottesville, Va.),8,5.1–23. 10.1167/8.11.5 1534-7362 18831599

[b25] Rolfs,M. (2009).Microsaccades: Small steps on a long way. Vision Research,49,2415–2441. 10.1016/j.visres.2009.08.010 0042-6989 19683016

[b11] Siegenthaler,E. , Costela,F. M. , McCamy,M. B. , Di Stasi,L. L. , Otero-Millan,J. , Sonderegger,A. , Groner,R. , Macknik,S. , & Martinez-Conde,S. (2014).Task difficulty in mental arithmetic affects microsaccadic rates and magnitudes. The European Journal of Neuroscience,39,287–294. 10.1111/ejn.12395 0953-816X 24438491

[b12] Valsecchi,M. , Betta,E. , & Turatto,M. (2007).Visual oddballs induce prolonged microsaccadic inhibition. Experimental Brain Research,177,196–208. 10.1007/s00221-006-0665-6 0014-4819 16951959

[b16] Watanabe,M. , Matsuo,Y. , Zha,L. , Munoz,D. P. , & Kobayashi,Y. (2013).Fixational saccades reflect volitional action preparation. Journal of Neurophysiology,110,522–535. 10.1152/jn.01096.2012 0022-3077 23636719

[b8] White,A. L. , & Rolfs,M. (2016).Oculomotor inhibition covaries with perceptual awareness. Journal of Neurophysiology,116,1507–1521. 10.1152/jn.00268.2016 0022-3077 27385794 PMC5040379

[b24] Zuber,B. L. , Stark,L. , & Cook,G. (1965).Microsaccades and the velocity-amplitude relationship for saccadic eye movements. Science,150,1459–1460. 10.1126/science.150.3702.1459 0036-8075 5855207

